# NGF and the Male Reproductive System: Potential Clinical Applications in Infertility

**DOI:** 10.3390/ijms232113127

**Published:** 2022-10-28

**Authors:** Giampiero Ferraguti, Francesca Fanfarillo, Luigi Tarani, Giovanna Blaconà, Francesca Tarani, Christian Barbato, Antonio Minni, Massimo Ralli, Silvia Francati, Antonio Greco, Carla Petrella, Marco Fiore

**Affiliations:** 1 Department of Experimental Medicine, Sapienza University of Rome, 00185 Rome, Italy; 2 Department of Maternal Infantile and Urological Sciences, Sapienza University of Rome, 00185 Rome, Italy; 3 Institute of Biochemistry and Cell Biology-National Research Council, IBBC—CNR, 00185 Rome, Italy; 4 Department of Sense Organs, Sapienza University of Rome, 00185 Rome, Italy

**Keywords:** male infertility, oligozoospermia, asthenozoospermia, azoospermia, sperm cryopreservation, assisted reproduction, nerve growth factor, neurotrophins

## Abstract

Infertility is a worldwide health issue defined by the World Health Organization (WHO) as the inability to establish a pregnancy after 12 months or more of regular and unprotected sexual intercourse. Male infertility etiology can be related to either congenital or acquired factors. The therapeutical approach to male infertility depends on the underlying causes and includes medical and surgical treatments. In recent studies, the potential role of nerve growth factor (NGF) in male reproductive physiology has been proposed. It has been hypothesized that neurotrophins might be involved in testis morphogenesis and regulation of several aspects of spermatogenesis. Moreover, it has been shown that NGF exerts its role on gonadotropin-releasing hormone (GnRH) neurons through the activation of the PKC/p–ERK1/2/p–CREB cascade, which leads to the activation of hypothalamic cells and the consequent activation of hypothalamus–pituitary–gonadal axis (HPG) with the secretion of GnRH. Lastly, it has been shown that the physiology of mature sperm is affected by both exogenous and endogenous NGF. The NGF impact on the HPG axis and its effect on GnRH neurons might be exploited in the therapy of male hypogonadism or used as a protective strategy against gonadal dysfunction related to chemotherapeutic agents. Moreover, the improving effect of NGF on sperm motility and vitality could be useful to enhance assisted reproduction outcomes. NGF could be supplemented to cryopreserved sperm samples to counteract the oxidative stress induced by the frozen and thawing processes. Indeed, the potential clinical applications of NGF in male infertility treatment have been discussed.

## 1. Introduction

During the past years, there has been growing interest in the understanding of male infertility causes and treatments. Infertility has an important social impact because it can affect mental health, being related to depression, anxiety disorders, and other psychological diseases. According to the WHO, the quality of life and psychological health depend directly on sexual health. Many factors can be related to male infertility, such as hormonal disease, obesity, diabetes, ejaculatory disorders, urogenital infections, testicular trauma, chemo/radiotherapy, or surgical treatments.

Recent findings reported the implication of nerve growth factor (NGF) in male reproductive pathophysiology. A wide number of animal and human studies demonstrated the involvement of NGF in spermatogenesis, testis morphogenesis, and the hypothalamus–pituitary–gonadal (HPG) axis and its improving effect on sperm traits. Therefore, NGF might be exploited in male infertility treatment, suggesting potential new strategies for male infertility therapy.

### 1.1. Male Infertility

#### 1.1.1. Epidemiology

Infertility is a worldwide health issue defined by the World Health Organization (WHO) as the inability to establish a pregnancy after 12 months or more of regular and unprotected sexual intercourse [1,2]. Infertility affects approximately 15% of reproductive-aged couples and 186 million individuals globally [3]. Data suggest that in about 50% of infertility cases, “male factor” infertility has an important role and is solely responsible in 20–30% of cases [4]. Infertility is related to psychological distress, leading to depression and anxiety disorders, and has an important social impact [5,6].

#### 1.1.2. Etiology

The etiology of male infertility can be related to a variety of factors, which can be distinguished into congenital or acquired [2]. Both congenital and acquired causes of male infertility can be classified into three categories: pretesticular, post-testicular, and testicular causes [7]. Among the pretesticular causes of male infertility, hypogonadotropic hypogonadism (HH) is one of the main causes [8]. Patients affected by HH have a deficit of LH and FSH secretion, which can be due either to a pituitary or hypothalamic dysfunction [9,10].

The hyposecretion of LH and FSH compromises normal spermatogenesis and production of testosterone, being responsible for infertility [11,12]. Other pretesticular causes of male infertility are coital disorders, such as ejaculatory disorders (e.g., anejaculation, retrograde ejaculation) [13,14] and erectile dysfunction [15]. It should be noted that some authors included coital disorders among the post-testicular causes of male infertility [16]. Post-testicular causes of male infertility also include primarily all the obstructions of the seminal tract [17,18]. Infections of the urogenital tract are also among the post-testicular causes of male infertility; in fact, microorganisms and leucocytes in the semen might damage sperm motility [19,20]. Moreover, activated leucocytes might produce reactive oxygen radicals (ROS), inducing sperm cell dysfunction [21].

Furthermore, inflammatory diseases of the accessory glands (e.g., prostatitis) and autoimmune reaction against the spermatozoa are included in the post-testicular causes of male infertility [22]. Post-testicular diseases might also be congenital, as in the case of CAVD (congenital absence of the vas deferens) [23]. Lastly, male infertility can be due to primitive testicular dysfunction, which causes impaired sperm production [24].

Testicular causes of male infertility include orchitis, testicular trauma, torsion, cryptorchidism (congenital or acquired) [25,26,27], systemic diseases, iatrogenic forms, and genetic abnormalities. Varicocele can be considered a cofactor of defective sperm production because it is associated with testicular atrophy and Leydig cell dysfunction [28]. In about 50% of cases, the etiology of male infertility still remains unknown (idiopathic infertility) [29]. Idiopathic infertility is probably, in most cases, related to genetic causes, considering that more than one thousand genes are involved in spermatogenesis [30].

#### 1.1.3. Treatments

The therapeutical approach to male infertility depends on the underlying causes and includes medical and surgical treatments [31]. Medical treatment of male infertility mainly involves hormonal treatment [32], which is based on the use of gonadotropin-releasing hormone (GnRH), gonadotropins, testosterone, dopamine agonists, aromatase inhibitors (AI), and selective estrogen receptor modulators (SERMs) [33].

Men affected by hypogonadotropic hypogonadism related to a reduced secretion of GnRH from the hypothalamus can be treated with GnRH therapy [34]. The pulsatile administration of GnRH stimulates the anterior pituitary to release gonadotropins [35], with the re-establishment of the hypothalamus–pituitary–gonadal (HPG) axis leading to high levels of testosterone and stimulation of Sertoli cells by the FSH [36]. In about 85% of patients treated with pulsatile GnRH, spermatogenesis is induced [37,38,39].

The administration of gonadotropins can be useful in men with hypogonadotropic hypogonadism related to pituitary dysfunction. It has been shown that gonadotropin therapy induces spermatogenesis in about 80% of patients [40,41]. Dopamine agonists are used for the treatment of male infertility associated with prolactin-secreting pituitary adenoma [42]. SERMs are indicated for the treatment of idiopathic infertility [43]. Their action is based on the inhibition of central estrogen feedback upregulating the production of pituitary gonadotropins [44]. Surgical therapy for male infertility is indicated mainly in men with obstructive azoospermia (OA) [45,46].

When treatment has failed or no specific treatment is available for the condition underlying male infertility, assisted reproductive technologies (ARTs) are indicated [47]. Among them, the most used and successful are intrauterine insemination (IUI), in vitro fertilization (IVF), and intracytoplasmic sperm injection (ICSI) [48,49].

IUI is a technique that involves the introduction of spermatozoa through the cervix using a catheter [50]. This technique is indicated in case of mild male infertility or unexplained infertility [51]. However, IUI needs a good semen quality and thus it is not fully indicated for “idiopathic oligozoospermia and asthenozoospermia or in men affected by retrograde ejaculation and anejaculation [51,52].

IVF facilitates fertilization by bringing the spermatozoa close to the oocyte [52] occurring outside the female body. IVF techniques usually include (i) a transvaginal ovum retrieval whereby a small needle is inserted through the back of the vagina and guided via ultrasound into the ovarian follicles to collect the fluid that contains the eggs and (ii) an embryo transfer whereby one or several embryos are placed into the uterus of the female with the intent to establish a pregnancy.

ICSI is the treatment of choice in case of IVF failure [53] and it consists of the injection of a single spermatozoon directly into the oocyte’s cytoplasm [54]. It can be used with ejaculated sperm, epididymal sperm, or testicular sperm. Retrieved epididymal or testicular spermatozoa are often cryopreserved to avoid repeated aspirations or biopsy in case of ART failure [55].

## 2. NGF

### 2.1. Neurotrophins

Neurotrophins are a family of growth factors mainly involved in the regulation of neuronal survival, function, and plasticity within the central and peripheral nervous systems. Neurotrophins include nerve growth factor (NGF), brain-derived neurotrophic factor (BDNF), neurotrophin-3 (NT-3), and neurotrophin-4 (NT-4) [56]. These factors exert their effects by binding two major receptor types: the p75 neurotrophin receptor (p75NTR) and the tropomyosin-related tyrosine kinase (Trk) receptors. P75NTR is a low-affinity receptor to which all neurotrophins can bind; neurotrophins, in the absence of Trk receptors, can bind to p75NTR, which acts as a death receptor.

Trk receptors are a family of three receptors including TrkA, which functions mainly as a receptor for NGF, TrkB (as a receptor for BDNF and NT-4), and TrkC (as a receptor for NT-3) [57]. Neurotrophins binding to Trk receptors activate different intracellular signaling cascades, including Ras/mitogen-activated protein kinase (MAPK), phosphoinositide 3-kinase/protein kinase B-mammalian target of rapamycin (PI3K/Akt-mTOR), and phosphoinositide-specific phospholipase C-γ (PLC-γ) pathways [58,59,60]. The activation of the Ras/MAPK and PI3K/Akt pathways is involved in promoting neuronal differentiation and survival [61,62]. PLC-γ pathways trigger the intracellular release of calcium ions from the endoplasmic reticulum, leading to the activation of calcium-dependent proteins and the expression of transcription factors and ion channels [63,64,65].

Among the neurotrophin family, nerve growth factor (NGF) was the first to be discovered. It was identified in the early 1950s by Rita Levi-Montalcini in mouse sarcoma cultures [66]. NGF shows neuroprotective and neurotrophic effects in the central nervous system and regulates the survival and maturation of developing neurons within the peripheral nervous system [67,68]. Moreover, NGF is involved in the neural response to damage in nociceptive sensory neurons, Schwann cells, and α motor neurons [69,70,71,72]. Neurotrophins are mainly known for their neurotrophic role, but they also exert a variety of effects outside the nervous system [73,74]. Changes in NGF in the serum and plasma were shown during the beginning and progression of many pathologies, including neurological, psychiatric and immune diseases [75,76], and physiopathological conditions, such as cardiometabolic disruptions [77,78], oxidant circumstances [79,80], stressful events [81,82], alcohol addiction [83,84,85], and aging [86,87,88].

Indeed, the expression of neurotrophin receptors in several non-neuronal tissues and the involvement of neurotrophins in essential non-neuronal functions, such as the maintenance of immune cells, has been demonstrated. In particular, NGF induces the differentiation of B lymphocytes [89] and is involved in the maintenance of neutrophils, peritoneal mast cells, and B lymphocytes [90,91,92].

### 2.2. NGF Expression in the Reproductive System

It is known that NGF plays a pivotal role in regulating neuronal cell growth and survival; increasing evidence shows that NGF also exerts a variety of effects on non-neuronal cells. In recent studies, the expression of NGF and its receptors (TrkA and p75NTR) outside the nervous system has been demonstrated, in particular in the male reproductive system [93,94,95,96], leading to the hypothesis that NGF could have a role in the reproductive system [97,98]. Moreover, pieces of evidence show that the testis and brain share a common embryonic origin [99,100], explaining the expression of neural receptors in sperm cells [101].

Ayer-LeLievre et al., in 1988, demonstrated that in testis and epididymis of rats and mice, NGF and TrkA were expressed [102]. Successively, a large body of studies confirmed these results. The presence of NGF was first detected in the prostate of guinea pigs [103], rabbits, and bulls [104]. In a study conducted on golden hamsters, it was found that NGF expression is greater in the caudal portion of the epididymis than in the other regions [105]. Many studies conducted on a variety of species, such as camelids, llamas, rabbits, and alpacas, demonstrated the presence of NGF and its receptors in seminal plasma [106,107,108,109,110]. In 2010, Li et al. reported the presence of NGF, TrkA, and p75NTR in the spermatozoa’s tail and head. Moreover, they showed that oligo/asthenozoospermic men had lower levels of NGF in their semen compared to fertile men [111]. All these findings suggest the potential role of NGF in male reproductive physiology [112,113].

## 3. NGF Specific Functions in the Male Reproductive System

### 3.1. NGF’s Role in Testis Morphogenesis

It has been demonstrated that neurotrophins are involved in the morphogenetic process regulating local cell–cell interactions in many tissues, such as kidney, tooth, dermatome, and ovary [114,115,116,117]. Moreover, neurotrophins play a role in germ cell survival and differentiation [118]. Therefore, it has been hypothesized that neurotrophins might be implicated in testis morphogenesis. The expression of neurotrophins in postnatal testis has been shown [102,119,120].

The expression of p75NTR in the mesenchymal tissue that surrounds the testis cord has been observed [121,122]. Levine et al. studied the effects of neurotrophins on seminiferous cord formation using the Trk-specific inhibitor K252a and the inhibitory TrkC-IgG antagonist [123]. They demonstrated that the inhibition of the neurotrophin pathway resulted in an inhibition of testis cord formation, suggesting that neurotrophins play a crucial role in testis morphogenesis [124].

Cupp et al. studied the role of TrkA and TrkC in the process of testis development, finding that TrkA is involved in the early stages of testis morphogenesis, whereas TrkC is involved in the later stages, both being implicated in the regulation of the germ cells number [125].

### 3.2. NGF Role in Spermatogenesis

Spermatogenesis involves a complex system of processes that lead to the production of mature spermatozoa in the seminiferous tubules (ST) [126,127]. The spermatogenic process requires three crucial steps: the mitotic division of spermatogonia [128]; meiosis I, with the generation of spermatocytes, and meiosis II, with the generation of spermatids [129]; and spermiogenesis, culminating in the production of mature spermatozoa [130]. Spermatogenesis is regulated by both endocrine and paracrine mechanisms [131,132].

The hormonal control is mediated by follicle-stimulating hormone (FSH) and testosterone [133]. The paracrine mechanism of regulation of spermatogenesis is mediated by Sertoli cells and germ cells [134], which are involved in the production of a variety of regulatory factors [135,136]. The proteins secreted by germ cells exert their effect on Sertoli cells, stimulating them to produce many molecules involved in the process of spermatogenesis, such as ABP [137], transferrin [138,139], IL-1a [140], SGP-2/1 [141], inhibin [142], and ceruloplasmin [143]. Moreover, germ cells inhibit the production of 17b-estradiol [144].

The process of spermatogenesis requires a synergic and/or redundant action of regulatory molecules in order to occur correctly [145,146]. Among these molecules involved in spermatogenesis regulation, there is growing attention on neurotrophins. The presence of different neurotrophins in mammalian testis has been demonstrated [147], but among them, the NGF is the only one that showed a potential role in spermatogenesis.

Many studies indicated the impact of NGF on several aspects of spermatogenesis. The presence of NGF protein [148] and mRNA [102] in germ cells has been shown, and its mitogen activity has been demonstrated [118]. NGF shows different effects, such as guaranteeing the physiological integrity of seminiferous epithelial cells [149], stimulating DNA synthesis within the seminiferous tubules [150], and inducing the secretion of ABP from Sertoli cells [120]. These effects are exerted through the binding between NGF and its receptors, p75NT and Trk [119,151], which are expressed on Sertoli cells.

The presence of NGF receptors on Sertoli cells in animals’ testis suggested the role of NGF in spermatogenesis regulation. Subsequently, the presence of NGF mRNA and p75NTR mRNA and protein in human testis was demonstrated [152,153].

### 3.3. The Impact of NGF on the Hypothalamus-Pituitary-Gonadal (HPG) Axis

Spermatogenesis is regulated by the hypothalamus–pituitary–gonadal (HPG) axis [154]. The hypothalamus produces gonadotropin-releasing hormone (GnRH) and releases it in a pulsatile manner [155]. The pulsatile secretion of neuropeptide is essential for stimulating the gonadotropic cells of the anterior pituitary to synthesize and secrete LH and FSH [156].

The stimulus of LH and FSH on testis cells activates two crucial endocrine signals: LH stimulates the production of testosterone from Leydig cells [157], and FSH induces the production of ABP (androgen-binding protein) and inhibin from Sertoli cells [158]. FSH and LH have a pivotal role in regulating spermatogenesis, mainly because they mediate the production of Sertoli factors, respectively, in a direct or indirect (through a testosterone–androgen receptor) way [159]. The HPG axis is finely regulated by a negative feedback mechanism [160].

**Figure 1:**
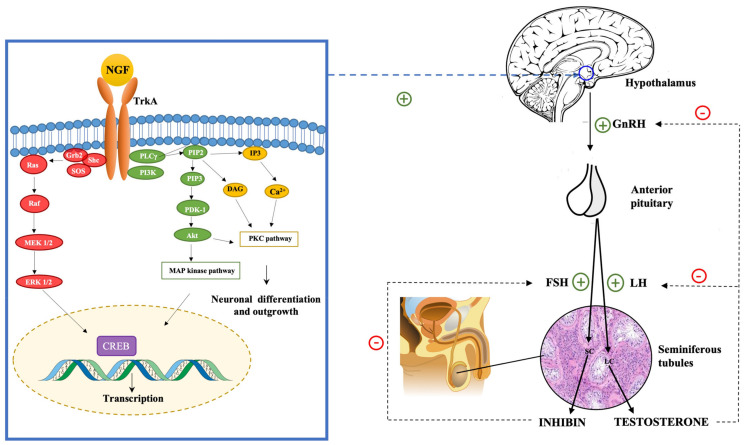
NGF regulates the hypothalamus–pituitary–gonadal (HPG) axis stimulating the hypothalamic production of GnRH. The binding of the NGF to the TrkA receptor activates many intracellular pathways, including the PI3Kinase pathway, which leads to the activation of Akt kinase, the Ras pathway, which leads to MAP kinase activation, and the PLC pathway, which leads to the activation of PKC. These intracellular pathways are involved in regulating the expression of neuronal survival and differentiation genes and the transcription of GnRH gene and neuropeptide production.

Testosterone, in high levels, inhibits the hypothalamic secretion of GnRH and the pituitary secretion of LH [161]. Inhibin acts on the anterior pituitary, inhibiting the production of FSH [162]. The NGF is involved in the regulation of the hypothalamus–pituitary axis [163,164]. Luo et al. in 2018 demonstrated that NGF can activate GnRH neurons in the hypothalamus and, thus, regulate the hypothalamic secretion of GnRH [165]. Moreover, they found that by using TrkA inhibitors, the effects of NGF on the HPG axis were extinguished. This finding led to the hypothesis that the impact of NGF on the hypothalamic secretion of GnRH was mediated by the TrkA receptor (see Figure 1).

The binding of NGF to TrkA activates many intracellular signaling cascades, such as MAPK, PI3K, and PLC-γ-PKC pathways [166]. GnRH transcription and neuropeptide production in GT1-7 cells are regulated by PKC, PKA, and MAPK pathways [167,168]. TrkA activation leads to the activation of ERK1/2 and ERK5. These kinases phosphorylate and activate CREB, Elk-1, and MEF2, which are transcription factors involved in regulating the expression of neuronal survival and differentiation genes [61,169]. The NGF exerts its role on GnRH neurons through the activation of the PKC/p-ERK1/2/p-CREB cascade, which leads to the activation of hypothalamic cells and the consequent activation of the HPG axis with the secretion of GnRH [170,171].

### 3.4. NGF Effects on Sperm Traits

**Figure 2:**
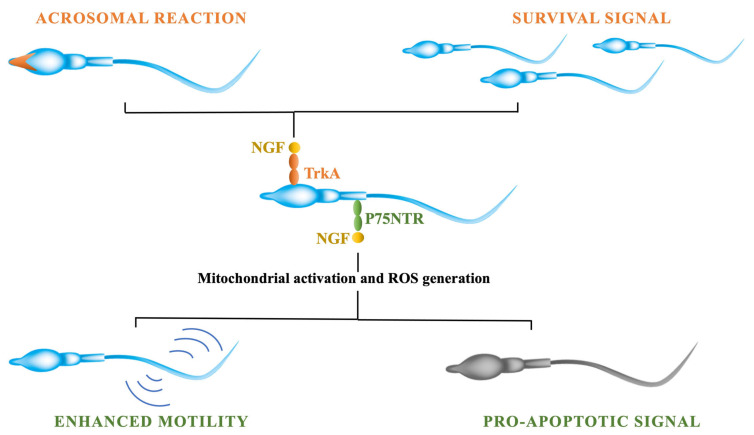
NGF exerts its effects on sperm cells through its binding to its two receptors: TrkA and p75NTR. The activation of the TrkA receptor is involved in stimulating cells’ survival and modulating the acrosomal reaction through the activation of the kinases’ pathway. The binding of the NGF to p75NTR receptor, instead, is involved in modulating sperm cells’ apoptosis by triggering the mitochondrial activity and the production of ROS. Moreover, the activation of p75NTR is involved in modulating sperm motility through the activation of the sperm’s respiratory chain.

Various studies demonstrated that nerve growth factor (NGF) exerts a variety of effects on mature sperm traits [172]. the expression of NGF receptors in sperm cells has been demonstrated: p75NTR is mainly in the midpiece and tail, whereas TrKA is expressed in the head and acrosome [173]. In 2010, two different studies reported the presence of NGF and the expression of TrkA receptor in animal sperm. One group studied hamster sperm, demonstrating that the NGF stimulates acrosome reaction and increases sperm motility (see Figure 2).

This stimulating effect of NGF on sperm motility was found to be time- and dose-dependent. The other group studied bovine sperm, demonstrating the presence of NGF and TrkA receptors in ejaculated sperm. They showed that exogenous NGF had positive effects on sperm viability and apoptosis, although the NGF did not affect acrosome reaction [174]. Two years later, a study on human sperm was published; it reported that NGF has an improving effect on human sperm motility traits, such as straight-line velocity, curvilinear velocity, average path velocity, linearity, and beat-cross frequency [175]. After incubating the sperm in vitro with NGF, the sperm motility increased in a dose-dependent manner [176]. Bezerra et al. studied the proteome of seminal plasma in rabbits and suggested that NGF expression might increase sperm motility [177].

According to the literature, NGF improves sperm motility and vigor [175,178]. Other studies showed that NGF also has an improving effect on the motility of cryopreserved sperm. It has been demonstrated that adding NGF in cryopreserved samples improves human sperm motility and viability, decreases apoptosis, and increases nitric oxide (NO) concentration [178]. Parthipan et al. compared the NGF levels in bovine sperm of “good” and “poor” donors and observed that NGF levels were higher in “good” sperm donors. Furthermore, they demonstrated that NGF exerts positive effects on sperm kinetic traits after thawing (e.g., sperm speed, linearity, straightness, and mitochondrial membrane potential) [179].

A recent study carried out on llamas suggests that the NGF contained in sperm has a seminal plasma origin; in fact, the NGF has been detected in ejaculated sperm, whereas it is not present in epididymal germ cells [180]. The NGF exerts its main effects on sperm traits through binding to its receptors, TrkA and p75NTR [181]. The activation of these receptors might also be involved in spermatogenesis and testicular development; in fact, the expression of NGF receptors in Leydig and Sertoli cells has been demonstrated [182].

The activation of the TrkA receptor leads to the activation of kinases (e.g., MAPK) that are involved in modulating the acrosomal reaction. Following the binding of the NGF to the TrkA, PI3kinase activation occurs, leading to an increase in cell survival [174]. The activation of p75NTR, instead, is important mainly in modulating capacitation, sperm motility, and apoptosis. It has been shown that by blocking p75NTR, the reduction of sperm track speed occurred. That was consistent with the high number of receptors expressed in the midpiece, which is the site of energy production by mitochondria. NGF at a 100 ng/mL dose significantly improved motility rate in comparison to lower doses; higher doses (125 and 150 ng/mL) did not further increased sperm motility [183]. The binding of the NGF to p75NTR activates the sperm’s respiratory chain, which is responsible for the sperm’s propulsive force [184]. As a consequence of this mitochondrial activation, the generation of reactive oxygen species (ROS) occurs [185,186]. Therefore, the activation of sperm motility and capacitation might be considered a preapoptotic status [187] associated with ROS generation [188]. The NGF is involved in modulating sperm apoptosis and capacitation by triggering mitochondrial activity and the production of ROS [189].

The NGF might have a pivotal role in modulating the survival and senescence of spermatozoa depending on which receptor pathway is activated: p75NTR proapoptotic or TrkA prosurvival. An in vitro study on rabbit sperm reported that the blocking of the TrKA resulted in a reduced number of motile cells, due to the positive impact of NGF–TrKA on the survival rate of sperm [189]. Two different P75NTR signaling pathways have been found: one including TrkA activation and the other focused only on P75NTR activity via NFkB activation [183]. Indeed, in the absence of TrkA, P75NTR can induce apoptosis [190]. These findings suggest that the role of NGF depends on the balance between its two receptors. The different “death–survival”pathways activated by NGF receptors’ interactions were determined by the concentration (ratio) of P75NTR to TrkA [191].

## 4. New Therapeutical Opportunities

### 4.1. The Potential Role of NGF in Male Infertility Treatment

Accumulating evidence has shown that NGF plays a role in the reproductive system [192,193,194,195,196]. Studies on Leydig cells’ development showed that NGF promotes the proliferation of stem Leydig cells (SLCs), progenitor Leydig cells (PLCs), and immature Leydig cells (ILCs) and induces PLCs to differentiate. These results suggested that the NGF pathway might be a potential target for the development of new therapies for diseases related to the dysfunction of Leydig cells, such as partial androgen deficiency of the aging male (PADAM) [197]. The standard treatment for testosterone deficiency syndrome, also known as hypogonadism, is testosterone supplementation therapy (TST) [198].

Exogenous testosterone can be administrated through several routes of delivery (e.g., nasal, buccal, subdermal, intradermal, and intramuscular), with the aim of increasing serum levels of testosterone and, thus, reducing signs and symptoms of hypogonadism [199]. However, testosterone therapy is related to several potential side effects. In fact, TST is not indicated in patients with a high risk of prostate cancer or important cardiovascular diseases [200]. Moreover, exogenous testosterone exerts inhibitory feedback on the HPG axis, resulting in the inhibition of intratesticular production of testosterone [201].

Alternative therapies are pulsatile GnRH administration and gonadotropin therapy, which are safer and more effective [202]. A further alternative therapeutic option for hypogonadism treatment might be represented by NGF administration. Luo et al. in 2018 studied the effects of NGF on hypogonadism. They demonstrated that NGF administration significantly increased serum levels of testosterone and gonadotropins, improved sperm quality, and restored fertility in aging mice [165]. NGF is able to exert these effects through the activation of GnRH neurons. Moreover, they suggested that the more convenient way to deliver NGF to the hypothalamus might be intranasal administration, associated with the liposome encapsulation of the protein [203,204,205]. In a successive study, published in 2021, Luo et al. showed that NGF administration could rescue spermatogenesis in azoospermic mice [206].

They induced azoospermia in mice by treating them with busulfan injections. Busulfan is a chemotherapy drug used to manage the allogeneic transplantation of hematopoietic progenitor cells in patients affected by chronic myeloid leukemia (CML) [207]. Busulfan administration is associated with gonadal dysfunction [208]. It has been shown that a single dose of busulfan injected in mice induced the alteration of testosterone and LH levels and destroyed differentiated spermatogonia, resulting in azoospermia [209,210]. In their study, Luo et al. showed that NGF nasal administration increased sexual hormone levels, enhanced sperm quality, and induced spermatogonia differentiation. NGF therapy resulted to restore spermatogenesis in azoospermic mice. These results suggest that NGF therapy might be used as a protective strategy against gonadal dysfunction related to busulfan treatment or other anticancer agents (see Table 1).

### 4.2. The Effect of NGF Addition to Cryopreserved Sperm

Cryopreservation of human semen is a procedure that can be useful in a variety of conditions [211]. Indeed, it may be useful before surgical treatment for infertility or before cytotoxic treatment for malignant diseases, such as radiotherapy or chemotherapy. Indeed, such therapies may lead to ejaculatory dysfunctions or testicular failure [212,213]; thus, sperm cryopreservation is an effective route to preserve male fertility [214]. Cryopreservation is used also for the storage of donors’ semen until the screening for infectious diseases confirms the negativity [215] (see Table 2).

Moreover, cryopreservation plays an important role in the management of male infertility [217]. It is used for the storage of sperm obtained from azoospermic men after testicular sperm extraction in order to avoid to repeat biopsies or aspirations [218]. The frozen semen can be used in assisted reproduction treatments, such as intrauterine insemination, in vitro fertilization, and ICSI [219,220,221]. However, cryopreservation can cause sperm cell damage. Both the processes of freezing and thawing expose spermatozoa to acute stresses [222] (see Table 2). A large number of studies were conducted to examine cryodamage in sperm cells [223,224,225]. It has been observed that spermatozoa after thawing showed a decreased motility and viability in comparison to the prefrozen state [226]. A correlation between the decrease of spermatozoa motility and mitochondrial damage after thawing has been demonstrated [227].

Cryopreservation can cause lipid peroxidation in the sperm membrane and induce an enhancement in reactive oxygen species concentration [228]. Elevated levels of reactive oxygen species (ROS) compromise sperm motility [229,230,231] and viability [232], DNA integrity [233], and acrosomal structure [234,235]. Cryopreservation leads to sperm apoptosis, affecting mitochondrial membrane properties and enhancing ROS generation [236] (see Table 2). It has been suggested that oxidative stress and apoptosis-inducing factors are responsible for DNA alteration in spermatozoa during the process of cryopreservation [237,238]. In order to protect sperm cells against cryodamage, different additives are used. The supplements used are mainly antioxidants, antifreeze proteins, and cryoprotectants [239,240].

Many studies have demonstrated that adding antioxidants to freezing samples might be useful to counteract ROS action and improve sperm functions [241]. It has been demonstrated that brain-derived neurotrophic factor (BDNF) supplementation to frozen-thawed human spermatozoa improves sperm parameters, showing a protective effect against oxidative stress and apoptosis [242]. It has been shown that NGF exerts an improving effect on cells’ viability [243]. Furthermore, it has been proposed that the addition of exogenous NGF to an incubation medium might improve sperm viability [174].

Among the sperm traits, one of the most important for fertilization is sperm motility. The oxidative stress induced by the cryopreservation process can lead to chromosomal and DNA damage, resulting in a decrease in sperm motility [244,245]. Supplementing frozen sperm with antioxidants, such as vitamin E and NGF, resulted in a significant enhancement of post-thaw motility [175,246]. Saeednia et al. studied the effects of exogenous NGF addition (0.5, 1, and 5 ng/mL) to cryopreserved semen samples. They showed that exogenous NGF significantly improved spermatozoa viability and motility, demonstrating the cryoprotective effect of NGF [178].

### 4.3. NGF Supplementation in Assisted Reproduction

The in vitro fertilizing capacity of spermatozoa is mainly correlated to two sperm parameters: vitality and progressive motility (PM) [247]. Both sperm traits are associated with an improved outcome of assisted reproductive technology, especially IVF and IUI, which require effective sperm motility [248]. Therefore, in order to increase the ART success rate, it might be useful to enhance sperm vitality and progressive motility [249,250,251]. A variety of studies were conducted to investigate the improving effect of different substances on sperm motility and vitality and thus their potential role as supplements to sperm processing media. It has been shown that caffeine, pentoxifylline, and 2-deoxyadenosine improve sperm motility [252], but they exert noxious effects on embryonic development [253].

Many authors suggested that sperm motility and vitality are positively affected by NGF, based on the detection of NGF and its receptors in spermatozoa and testis [254]. In a recent study, it has been demonstrated that supplementing IVF medium with NGF could enhance embryonic cleavage rates and blastocyst hatching rates in bovines, leading to an improvement in IVF outcomes. Nevertheless, in this study both the spermatozoa and the oocytes were exposed to NGF, and the authors suggested that NGF can act on the oocyte in a direct way [255]. Subsequently, in 2021, the improving effects of IGF-I and NGF on human spermatozoa’s motility and vitality were studied. In this study, the authors showed that incubating spermatozoa in a medium supplemented with IGF-I or NGF can enhance in vitro vitality and progressive motility of spermatozoa, suggesting their potential role in improving assisted reproduction outcomes [216].

## 5. Conclusions

In conclusion, the latest studies have shown that neurotrophins, especially NGF, are implicated in the male reproductive system. Some studies were conducted to investigate the possible role of NGF in the management of male infertility. Indeed, it has been demonstrated that NGF has an improving effect on male infertility in aging mice and is able to restore fertility in male mice with busulfan-induced infertility. Moreover, some studies showed the potential application of NGF in ARTs. In fact, NGF might be supplemented to IVF medium in order to improve IVF outcomes. In addition, it could be used as an antioxidant supplement for cryopreserved semen samples in order to reduce sperm cells’ cryodamage. Further studies are required to reach a better understanding of the involvement of NGF in male reproductive system pathophysiology and, hence, its potential role in the management of male infertility.

## Figures and Tables

**Table 1:** Potential NGF therapeutical applications in male infertility.

Ref.	Methods	Results	Potential Clinical Applications
[197]	The effects of NGF on Leydig-cell (LC) regeneration were investigated by measuring mRNA levels in the adult rat testis after ethane dimethanesulfonate (EDS) treatment. The established organ culture model of rat seminiferous tubules was used to examine the regulation of NGF during SLC proliferation and differentiation using EdU staining, real-time PCR and Western blotting. Progenitor Leydig cells (PLCs) and immature Leydig cells (ILCs) were also used to investigate the effects of NGF on LCs at different developmental stages.	NGF significantly promoted SLCs proliferation with an obvious dose–response relationship. There is strong evidence that NGF can induce SLCs to differentiate into LCs.	NGF pathway might be a potential target for the development of new therapies for diseases related to the dysfunction of Leydig cells, such as Partial Androgen Deficiency of the Aging Male (PADAM).
[165]	The ten-month-old aging male senescence accelerate mouse P8 (SAMP8) mice with age-related hypogonadotropic hypogonadism were used to study the role of NGF in hypogonadism. The age-matched accelerated senescence-resistant mouse R1 (SAMR1) served as a control. The ten-month-old SAMP8 mice were treated with NGF twice per week for 12 weeks. Sexual hormones, sexual behaviors, and fertility were analyzed after NGF treatment. The mechanisms of NGF in sex hormones’ sexual function were also studied.	NGF could enhance the sexual function, improve the quality of the sperm, and restore the fertility of aging male SAMP8 mice with age-related hypogonadism by activating gonadotropin-releasing hormone (GnRH) neurons and regulating the secretion of GnRH. NGF regulated the GnRH release through the PKC/p-ERK1/2/p-CREB signal pathway.	NGF administration might represent an alternative therapeutic option for hypogonadism treatment.
[206]	A model with azoospermia induced by a single intraperitoneal injection of busulfan was established. NGF pre-encapsulated with liposomes (25, 50, and 100 μg/kg) was delivered via internasal administration. Three weeks after busulfan injection, NGF treatments were performed twice a week for 8 weeks; the change in sperm quality, testis and epididymis histopathology, and androgenic hormone were analyzed to evaluate sperm regeneration.	NGF with nasal administration could significantly upregulate the markers expressing meiotic spermatogonia (Stra8) and spermatocytes (SYCP3), restore spermatogenesis, and improve sperm quality.	NGF therapy might be used as a protective strategy against gonadal dysfunction related to busulfan treatment or other anticancer agents.

**Table 2:** Potential NGF application in semen preservation.

Ref.	Methods	Results	Potential Clinical Applications
[178]	Semen samples were collected from 25 normozoospermic men and were divided into fresh semen samples as the control group, frozen–thawed semen samples without the addition of exogenous NGF, and three groups of semen samples cryopreserved with addition of exogenous NGF (0.5, 1, and 5 ng/mL) in freezing medium. Viability, motility, and NO concentration were evaluated.	Results showed that exogenous NGF at 0.5 ng/mL could significantly (*p*-value < 0.05) influence viability, motility, nitric oxide, and DNA fragmentation content.	NGF supplementation to cryopreserved semen might be useful to improve spermatozoa viability and motility and reduce cells’ cryodamage.
[216]	Forty-three volunteers gave semen samples after 2–3 days of sexual abstinence. Each sample was processed with density gradient centrifugation and sperm washing. The pellet was divided into 3 aliquots. An aliquot containing one million progressively motile spermatozoa was incubated for an hour (37 °C) in a standard culture medium (control group), and two aliquots with the same number of progressively motile spermatozoa were incubated in a medium supplemented with IGF-I or NGFβ. Two concentrations of IGF-I (100 ng/mL and 1000 ng/mL) and NGFβ (0.5 ng/mL and 5 ng/mL) were tested.	Both growth factors significantly increased PM and vitality in comparison with control either at the low or the high concentration. IGF-I seemed to be more effective than NGFβ. The effects did not seem to be dose-dependent, with the exception of the effect of IGF-I on vitality.	Incubating spermatozoa in a medium supplemented with IGF-I or NGF can enhance in vitro vitality and progressive motility of spermatozoa, suggesting their potential role in improving assisted reproduction outcomes.

## Data Availability

Not applicable.

## Abbreviations

ABP	androgen-binding protein
AKT	protein kinase B
ARTs	assisted reproductive technologies
BDNF	brain-derived neurotrophic factor
CAVD	congenital absence of the vas deferens
CML	chronic myeloid leukemia
CREB	c-AMP-response element binding protein
DAG	diacylglycerol
EdU	5-ethynyl-2′-deoxyuridine
ERK	extracellular signal-regulated kinase
FSH	follicle-stimulating hormone
GnRH	gonadotropin-releasing hormone
GRB2	growth factor receptor-bound protein 2
HH	hypogonadotropic hypogonadism
HPG	hypothalamus–pituitary–gonadal
ICSI	intracytoplasmic sperm injection
ILCs	immature Leydig cells
IP3	inositol triphosphate
IUI	intrauterine insemination
IVF	in vitro fertilization
LC	Leydig cell
LH	luteinizing hormone
MAPK	mitogen-activated protein kinase
MEK	mitogen-activated protein kinase
NGF	nerve growth factor
NO	nitric oxide
NT-3	neurotrophin-3
NT-4	neurotrophin-4
OA	obstructive azoospermia
PADAM	partial androgen deficiency of the aging male
PDK	3-phosphoinositide-dependent kinase 1
PI3K	phosphoinositide 3-kinase
PIP2	phosphatidylinositol 4,5-bisphosphate
PIP3	phosphatidylinositol 3,4,5-trisphosphate
PKC	protein kinase C
PLC-γ	phosphoinositide-specific phospholipase C-γ
PLCs	progenitor Leydig cells
PM	progressive motility
RAF	rapidly accelerated fibrosarcoma
ROS	reactive oxygen species
SC	Sertoli cell
SERMs	selective estrogen receptor modulators
SHC	src honology and collagen
SLCs	stem Leydig cells
TST	testosterone supplementation therapy
WHO	World Health Organization
